# Prevalence of Self-Reported Pain, Joint Complaints and Knee or Hip Complaints in Adults Aged ≥ 40 Years: A Cross-Sectional Survey in Herne, Germany

**DOI:** 10.1371/journal.pone.0060753

**Published:** 2013-04-30

**Authors:** Ulrich Thiem, Rainer Lamsfuß, Sven Günther, Jochen Schumacher, Christian Bäker, Heinz G. Endres, Josef Zacher, Gerd R. Burmester, Ludger Pientka

**Affiliations:** 1 Department of Geriatrics, University of Bochum, Marienhospital Herne, Herne, Germany; 2 Department of Medical Informatics, Biometry and Epidemiology, University of Bochum, Bochum, Germany; 3 Department of Internal Medicine, Alfried Krupp Krankenhaus Steele, University of Duisburg-Essen, Essen, Germany; 4 Department of Orthopedics, HELIOS Klinikum Berlin-Buch, Berlin, Germany; 5 Department of Rheumatology and Clinical Immunology, Charite Berlin, University of Berlin, Berlin, Germany; Tehran University of Medical Sciences, Iran (Islamic Republic of)

## Abstract

**Background:**

Pain and musculoskeletal complaints are among the most common symptoms in the general population. Despite their epidemiological, clinical and health economic importance, prevalence data on pain and musculoskeletal complaints for Germany are scarce.

**Methods:**

A cross-sectional survey of a random sample of citizens of Herne, Germany, aged ≥ 40 years was performed. A detailed self-complete postal questionnaire was used, followed by a short reminder questionnaire and telephone contacts for those not responding. The questionnaire contained 66 items, mainly addressing pain of any site, musculoskeletal complaints of any site and of knee and hip, pain intensities, the Western Ontario MacMaster Universities (WOMAC) index, medication, health care utilization, comorbidities, and quality of life.

**Results:**

The response rate was 57.8% (4,527 of 7,828 individuals). Survey participants were on average 1.3 years older, and the proportion of women among responders tended to be greater than in the population sample. There was no age difference between the population sample and 2,221 participants filling out the detailed questionnaire. The following standardized prevalences were assessed: current pain: 59.7%, pain within the past four weeks: 74.5%, current joint complaints: 49.3%, joint complaints within the past four weeks and twelve month: 62.8% and 67.4%, respectively, knee as the site predominantly affected: 30.9%, knee bilateral: 9.7%, hip: 15.2%, hip bilateral: 3.5%, knee and hip: 5.5%. Pain and musculoskeletal complaints were significantly more often reported by women. A typical relationship of pain and joint complaints to age could be found, i.e. increasing prevalences with increasing age categories, with a drop in the highest age groups. In general, pain and joint pain were associated with comorbidity and body mass index as well as quality of life.

**Conclusions:**

Our data confirm findings of other recent national as well as European surveys. The high site specific prevalences of knee and hip complaints underline the necessity to further investigate characteristics and consequences of pain and symptomatic osteoarthritis of these joints in adults in Germany.

## Introduction

Musculoskeletal complaints are among the most prevalent symptoms in the general population. Recent European studies report prevalences between 30% and 80% [1]–[5]. The major cause of musculoskeletal pain is osteoarthritis [6]–[8]. Osteoarthritis is consistently related to age and female sex. To a different extent determined by disease severity and the site affected, osteoarthritis results in impairments of function, activities of daily living and quality of life [1], [4], [9]–[11]. Clinically, pain and symptomatic osteoarthritis of knee and hip are of special concern, as resulting functional impairments of these joints frequently compromise every day function and independence of living [1], [6], [9], [12], [13].

Musculoskeletal pain and osteoarthritis are also associated with a high economic burden [14]–[16]. In Germany, musculoskeletal diseases accounted for 26,6 billion Euro or 11.3% of the total health expenditures in 2006. Osteoarthritis caused approximately 7,1 billion Euro of direct costs, or roughly 3.3% of expenditures [15]. Due to the demographic change and an increasing prevalence of musculoskeletal conditions and osteoarthritis, an increase in the economic burden is expected for Germany as well as other industrialised countries [8], [14], [16].

Despite their epidemiological, clinical and health economic importance, current data on the prevalence of pain, musculoskeletal complaints and osteoarthritis in Germany are scarce. The Robert Koch Institute, the central federal institute for disease control and prevention in Germany, provides data from its 1998 National Health Interview and Examination Survey (BGS98) [11], [17], [18]. The BGS98 reported a period prevalence for musculoskeletal pain during the preceding seven days of up to 40% for the general adult population, and a yearly pain prevalence of up to 60%. A life time prevalence of self-reported, physician diagnosed osteoarthritis of 27.7% has also been estimated. Pain prevalences were associated with female sex and higher age [17], [18]. A drawback of the BGS98 is that neither point estimate for actual pain nor the pain site knee has been assessed. A regional extension of the BGS98 in the federal state of Bavaria [19] estimates a life time prevalence of osteoarthritis diagnosed by a physician of 18%, with no further estimate for pain.

In a survey by Gunzelmann et al. on pain in older adults aged 60 years and above, a pain prevalence of more than 80% has been reported [20]. This survey did not reveal differences by age or sex. A local telephone survey performed by our study group [21] assessed prevalences for current musculoskeletal pain and pain within the past four weeks and twelve month of 37.4%, 53.0% and 60.0%, respectively, and a prevalence of physician diagnosed osteoarthritis of 27.4%.

Earlier German studies (before year 2000) reported prevalences of any pain and joint complaints as well between 40% and more than 80% [22]–[25]. Site specific prevalences for hip or leg of approximately 7% to 19% and 18% to 57% were estimated, respectively [23], [25]. Again, prevalences for knee complaints were not assessed.

Highlighting the contrast between the clinical and economic importance of musculoskeletal pain and osteoarthritis and the rather small data base in Germany, a recent health economic review encouraged further national surveys and studies in the epidemiology and consequences of musculoskeletal conditions [16]. For this reason, we performed a cross-sectional survey on pain, musculoskeletal complaints and osteoarthritis in Herne, Germany.

## Methods

### Study aims

The aims of this study are to estimate

1) the prevalence of any pain,

2) the prevalence of musculoskeletal complaints, and

3) the site specific prevalence of musculoskeletal complaints for knee and hip in a population-based sample of adults aged 40 years and above.

### Study design

For these aims, we designed a cross-sectional postal survey using a random sample of citizens of Herne, Germany. With 171,831 registered inhabitants in 2005, 54.8% being 40 years of age and older, Herne belongs to the greater cities (population of more than 100,000 citizens) in Nordrhein-Westfalen (NRW), one the federal states in the west of Germany. In terms of age and sex distribution, the population of Herne is representative for all 23 greater cities in Nordrhein-Westfalen, covering an urban population aged 40 years and above of roughly 7,4 million people (data from the Federal Institute for Statistics NRW).

The city's Office of Statistics and Election provided a random sample of 8,000 registered citizens of Herne aged 40 years and above. The data set contained name, zip code, street, street number, year of birth and sex. 172 cases had to be excluded due to death or change of residence of the person or errors of the registry. The resulting basic sample contained 7,828 persons, or roughly every 22^nd^ adult citizen of Herne aged 40 years or older.

The study was made public by means of newspaper and media reports and information of local general practitioners, orthopaedic specialists and physicians of further specialties. A public event taking place four days before the first mailing was organized with talks on different aspects of musculoskeletal disorders and pain and information desks of the study team as well as different local health care providers, health maintenance organisations and self-help groups. End of February 2005, a detailed self-complete questionnaire was sent out to every person of the sample accompanied by a freepost return envelope and a letter informing about the aims of the study and inviting to participate. A short reminder questionnaire was mailed to every person not responding within the following four weeks. For those who did not respond to either of the questionnaires, attempts were made to contact them by phone.

### Survey questionnaire

The survey questionnaire comprised 20 pages. On the front page, name, address, date of birth and sex were assessed, and written informed consent for survey participation was gathered. The last page allowed remarks of the participants. The main questionnaire contained 66 questions and gathered data on overall and musculoskeletal pain, joint complaints, the predominant site of musculoskeletal complaints, pain intensities by means of 11-categorial visual-analogue-scales (VAS), the German version of the Western Ontario MacMaster Universities (WOMAC) Index [26], [27], actual medication with an emphasis on pain medication, number of physician visits and hospital stays due to musculoskeletal pain in the preceding twelve month, comorbidities, height, weight, self-perceived general health and quality of life.

### Questions on pain, joint complaints and predominant site of joint complaints

The questions on pain and complaints addressed the day the questionnaire was answered (for point prevalences) and the preceding four weeks and twelve month (for period prevalences). The questions for overall pain were: “Do you have pain in any part of your body today?” and “Did you have pain in any part of your body during the past four weeks?”. The questions on musculoskeletal pain and complaints were: “Do you have joint complaints today?” and “Did you have joint complaints during the past four weeks/twelve month?”.

Different joints or body regions were offered to localise complaints, namely shoulder, elbow, hand/fingers, hip, knee, ankle, foot/toes, upper and lower back. Participants were also asked whether the predominant site of complaints were knee, hip or another site. A further question was which disease was cause of pain or complaints at the predominant site. Several options were offered including osteoarthritis (“wear and tear”), rheumatoid arthritis, fibromyalgia and gout.

### Reminder questionnaire and telephone interview

The reminder questionnaire and the telephone interview contained the five prevalence questions on pain and joint complaints and the one on the predominant site of joint complaints, using the same wording as in the survey questionnaire. The survey questionnaire was offered and sent out again on request. Two trained interviewers mainly performed the telephone interviews, making several attempts to contact non-responders at different days and times during the day, usually within a ten day period.

### Sample size estimation and statistical analysis

We calculated 95% confidence intervals [95% CI] for different prevalences and a varying number of participants, considering a width of the 95% CI of±1.5% precise enough for the analysis of the whole sample, and a width of±3.5% for subgroups. This is achieved for prevalences between 10% and 90% as long as 4,000 or 800 individuals, respectively, participate.

Prevalences are reported crude and standardized for the age and sex distribution of the population of Herne, as well as categorised in 5 year age groups. The denominator of proportions varies by roughly±1% due to missing values. As a measure of association, we calculated odds ratios (OR) for categorial variables, also standardized for the Herne population. Tests of significance were performed at a level of α = 0.05, using the χ^2^-test for categorical and Student's t-test for continuous variables.

The calculation of confidence intervals for the sample size estimation and for proportions in the survey analysis was performed with CIA software (Bryant T., Confidence Interval Analysis, version 2.1.2., 2004). For all other analysis we used SPSS 14.0 (SPSS Inc., 2005).

### Ethics

The survey reported here was the first part of a larger project on musculoskeletal pain and osteoarthritis in Herne, Germany. The second part of the project was a cross-sectional study with a single clinical visit free of charge for survey participants reporting knee or hip pain or osteoarthritis and willing to participate. For both the conduct of the project and the analysis of survey data, ethics approval was obtained from the institutional review board of the University of Bochum, Germany.

The cover page of the survey questionnaire contained a consent statement that had to be signed by participants. Only questionnaires with a signed consent statement were considered. Participants providing information by telephone interview gave verbal consent at the beginning of the interview. If verbal consent was given, the interviewer began with the survey questions documenting the answers via an entry mask into an electronic database. In case the person phoned did not agree, the interviewer pressed a button on the data entry mask that coded a "non-participation" variable. This variable confirmed that the person was reached by phone, but declined participation, thus preventing that the person was contacted again. All data for this study was analyzed anonymously.

## Results

### Response

**Figure 1:**
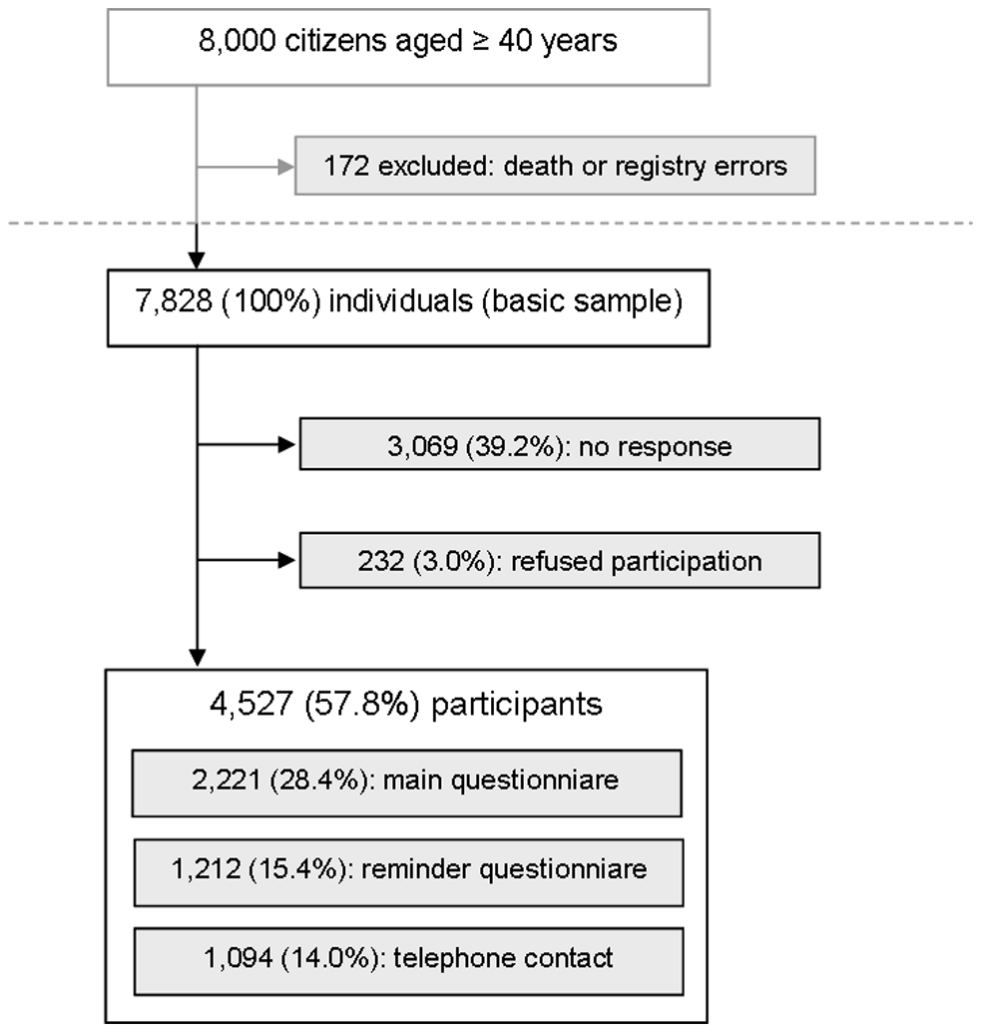
Survey profile and response.

An overview over the survey and the response categories is given in figure 1. In total 4,527 adults participated, resulting in a response rate of 57.8%. 3,069 individuals (39.2%) were non-responders. 38 of these, asked for their reasons not to participate, mentioned lack of time, no interest in the issue and the length of the questionnaire as the three main reasons for non-participation. 232 persons (3.0%) actively refused participation, either by mail or by phone call.

In the basic sample of 7,828 individuals, the mean age was 60.9 years (95%-CI [60.6; 61.2], median: 59.9 years), and women were on average 3.6 years (95%-CI [3.1; 4.3]) older than men. Among the 4,527 participants, the mean age was 1.3 years higher (62.2, 95%-CI [61.8; 62.6], median: 62.5 years), and women were on average 1.8 years (95%-CI [1.1; 2.6]) older than men. There was a trend to a higher proportion of women among responders (56.2%, 95%-CI [54.8; 57.7]) in comparison to the basic sample (54.0%, 95%-CI [52.9; 55.1]).

Of 4,527 participants, 2,221 filled out the detailed main questionnaire. These participants had a mean age of 60.4 years (95%-CI [59.9; 60.9], median: 60.2). In this subsample, no age difference between women (mean 60.7, 95%-CI [60.0; 61.4]) and men (mean 60.1, 95%-CI [59.3; 60.8]) was found. A trend towards a higher proportion of women (56.4%, 95%-CI [54.3; 58.5]) as compared to the basic sample could be observed.

Women were significantly more likely to actively refuse participation in comparison to men (p<0.001). People refusing actively were significantly older than responders and non-responders (mean age 70.6 years, 95%-CI [68.8; 72.4]).

**Figure 2:**
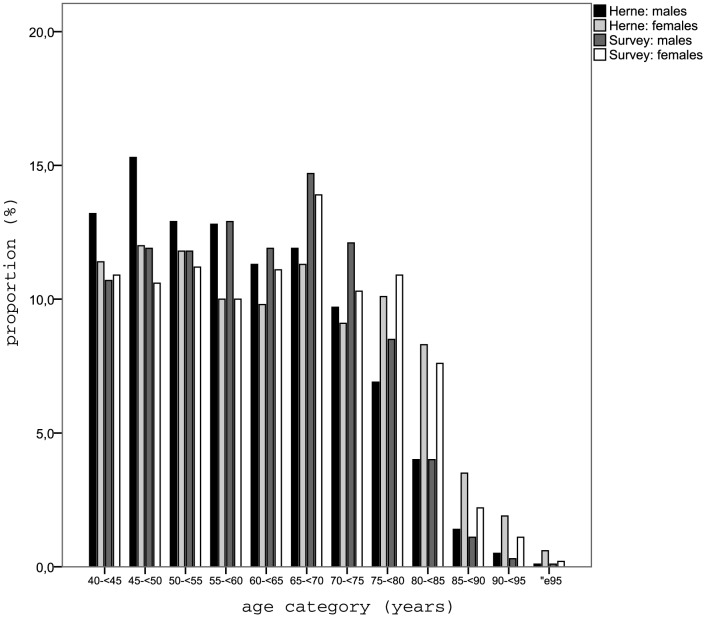
Distribution of age and sex in the population of Herne and among survey participants.

The distribution of age and sex among survey participants in comparison to the baseline sample can be seen in figure 2. For both sexes, the younger age categories between 40 and less than 55 years of age were less represented among participants, as were the oldest age groups above 85 years. The most pronounced difference to the disadvantage of survey participants was found in the age category of 45 to less than 50 years, with a difference of 3.4% for men and 1.4% for women, respectively. By contrast, the age groups between 55 and less than 80 years of age were overrepresented in both men and women, with the largest differences (2.8% for men, 2.6% for women) found for the age category of 65 to less than 70 years.

For the subsample of participants answering the detailed questionnaire, the picture was slightly different (data not shown). For men, the younger age groups and those of the oldest participants were underrepresented again, with the greatest difference being 3.0% in the age category 45 to less than 50 years of age. Among male participants, the age categories between 50 and less than 80 years were overrepresented, with a maximal difference of 2.5% in the category of 65 to less than 70 years. By contrast, in women all age groups under age 75 years were overrepresented in participants, the largest difference being 2.6% for the category of age 60 to less than 65 years. With 3.1%, the greatest difference to the disadvantage of female participants was that in the age category of 80 to less than 85 years.

### Prevalence data

The prevalences for pain and joint complaints for different time periods and those for knee and hip as the predominant site affected are listed in table 1. Approximately 60% reported any current pain, and approximately 50% current joint complaints. About 30% described the knee as the joint predominantly affected, about 15% the hip. More than 5% named both knee and hip as equally affected.

**Table 1:** Prevalence of any pain, joint complaints and knee and hip complaints.

	all participants		women		men	
	crude	stand.*	crude	stand. *	crude	stand. *
pain						
current	60.4%	59.7%	63.2%	62.6%	56.8%	56.2%
within the last four weeks	74.9%	74.5%	77.5%	77.0%	71.6%	71.4%
joint complaints						
current	50.0%	49.3%	52.4%	51.8%	47.0%	46.4%
within the last four weeks	63.4%	62.8%	66.6%	66.0%	59.4%	59.0%
within the last twelve months	68.0%	67.4%	70.9%	70.3%	64.2%	64.1%
predominant site						
any knee	31.4%	30.9%	30.9%	30.6%	31.9%	31.6%
*knee both sides*	*9.9%*	*9.7%*	*9.6%*	*9.5%*	*10.2%*	*10.2%*
any hip	15.6%	15.2%	15.9%	15.8%	15.3%	14.6%
*hip both sides*	*3.5%*	*3.5%*	*2.8%*	*2.9%*	*4.4%*	*4.3%*
*knee & hip*	*5.7%*	*5.5%*	*5.7%*	*5.8%*	*5.6%*	*5.3%*

* stand.: standardized for the population of Herne (reference day: 2004, December 31).

For elderly people aged 60 years and above, the following standardised prevalences were estimated: 64.1% and 76.9% for current pain and pain within the last four weeks, 54.9%, 66.9% and 70.6% for joint complaints currently and within the last four weeks and twelve months, respectively. For knee and hip we assessed: any knee 35.7%, knee bilateral 11.3%, any hip 19.2%, hip bilateral 4.0%, and knee and hip concomitantly 7.6%.

When all non-respondents and refusals were accounted for as free of pain or joint complaints, the following conservative estimates standardized for the Herne population resulted: 34.9% and 43.3% for current pain and pain within the last four weeks, 28.9%, 36.7% and 39.4% for joint complaints currently and within the last four weeks and twelve months, respectively. For knee and hip we calculated: any knee 18.2%, knee bilateral 5.7%, any hip 9.1%, hip bilateral 2.0%, and knee and hip concomitantly 3.3%.

### Associations with sex and age

Table 2 shows odds ratios for female sex and standardised prevalences. For all pain and complaint prevalences, a significantly increased risk for women was detected, with odds ratios ranging from 1.25 to 1.35. For knee or hip complaints, no clear sex difference could be found.

**Table 2:** Pain, joint complaints, knee and hip complaints and female sex.

	Odds ratio	95% CI*		
pain				
current	1.31	[1.21	;	1.42]
within the last four weeks	1.35	[1.23	;	1.47]
joint complaints				
current	1.25	[1.15	;	1.35]
within the last four weeks	1.35	[1.25	;	1.47]
within the last twelve months	1.32	[1.22	;	1.44]
predominant site				
knee	0.95	[0.87	;	1.03]
knee both sides	0.92	[0.81	;	1.05]
hip	1.10	[0.99	;	1.23]
hip both sides	0.67	[0.54	;	0.83]
knee & hip	1.10	[0.93	;	1.31]

* 95% CI: 95% confidence interval. Odds ratios calculated for standardized prevalences.

In tables 3 and 4, proportions of current pain and current joint complaints are shown by sex and age categories. For any pain in women, an increase of pain with increasing age could be observed, peaking in the age category of 70 to less than 75 years. A decrease in pain was evident in the oldest age groups only, i.e. above 90 years. For men, a comparable relationship was found, although less pronounced. The proportion reporting pain was higher in the youngest age group, but peaked at a lower level at age 70 to less than 75 years of age (table 3).

**Table 3:** Current pain by age categories and sex.

age	*current pain*					
	total		men		women	
years	n	%	n	%	n	%
40–44	232	47.4	110	52.1	122	43.9
45–49	262	52.0	121	51.5	141	52.4
50–54	314	60.7	129	55.6	185	64.9
55–59	310	61.0	145	56.9	165	65.2
60–64	331	64.0	138	58.7	193	68.4
65–69	400	62.9	162	56.6	238	68.0
70–74	341	68.3	151	63.4	190	72.8
75–79	280	63.5	101	61.2	179	64.9
80–84	180	66.9	48	61.5	132	69.1
85–89	49	62.0	8	36.4	41	71.9
90–94	17	53.1	2	40.0	15	55.6
> 95	1	14.3	0	0.0	1	16.7
total	2,717	60.4	1,115	56.8	1,602	63.2

χ^2^-test: total: p<0.001, men: p>0.05, women: p<0.001.

**Table 4:** Current joint complaints by age categories and sex.

age	*current joints complaints*					
	total		men		women	
years	n	%	n	%	n	%
40–44	165	33.7	85	40.3	80	28.8
45–49	200	39.7	98	41.7	102	37.8
50–54	265	51.3	109	47.0	156	54.8
55–59	254	50.0	118	46.3	136	53.8
60–64	282	54.6	118	50.2	164	58.2
65–69	347	54.6	145	50.7	202	57.7
70–74	281	56.3	122	51.3	159	60.9
75–79	239	54.2	83	50.3	156	56.5
80–84	154	57.3	38	48.7	116	60.7
85–89	42	53.2	7	31.8	35	61.4
90–94	16	50.0	2	40.0	14	51.9
> 95	1	14.3	0	0.0	1	16.7
total	2,246	50.0	925	47.1	1,321	52.1

χ^2^-test: total: p<0.001, men: p>0.05, women: p<0.001.

For musculoskeletal complaints in women, a typical relationship with age could be observed. An increase with higher age categories was found, with a prevalence of more than 60% in the age category 85 to less than 90 years. After this, a decline of the prevalence could be seen. For men again, the trend was weaker, with a plateau of the prevalence around 50% for ages between 60 and 80 years (table 4).

We also assessed the prevalence of current pain unrelated to joint complaints by calculating proportions of participants who reported any current pain without simultaneously reporting musculoskeletal complaints (data not shown). For this group, a reversed age dependency was found. For women, the highest proportion (14.4%) was seen in the youngest age category, i.e. 40 to less than 45 years of age. With 11.5%, a further peak could be observed for age 70 to less than 75 years. After either peak, the prevalence was decreasing with increasing age category. A similar pattern could be found for men, although less pronounced, especially in the younger age groups.

Table 5 shows proportions of participants describing the knee as the predominant site of joint complaints by sex and age categories. The relationship of knee complaints and age follows a pattern already found for any current pain and current joint complaints of any site.

**Table 5:** Knee complaints by age categories and sex.

age	*knee complaints*					
	total		men		women	
years	n	%	n	%	n	%
40–44	110	22.5	63	29.9	47	16.9
45–49	108	21.4	66	28.1	42	15.6
50–54	156	30.1	75	32.2	81	28.4
55–59	152	29.7	71	27.6	81	31.8
60–64	177	34.0	82	34.5	95	33.7
65–69	211	32.6	84	28.7	127	35.9
70–74	180	35.9	88	36.8	92	35.1
75–79	173	38.9	63	37.5	110	39.7
80–84	106	39.0	29	36.7	77	39.9
85–89	30	38.0	7	31.8	23	40.4
90–94	14	43.8	3	60.0	11	40.7
> 95	1	14.3	0	0.0	1	16.7
total	1,418	31.3	631	31.9	787	30.9

χ^2^-test: total: p<0.001, men: p>0.05, women: p<0.001.

### Associations with other variables

For respondents to the main questionnaire, we were able to investigate the association between pain or joint complaints and some further variables. Respondents to the main questionnaire reporting current pain at any site were significantly older than those without pain (mean age 61.5 years±11.9 years standard deviation versus 57.7±12.2 years, p<0.001) and more often female (p = 0.001). They had significantly higher BMI values as calculated from self-reported weight and height than participants without pain (mean difference: 1.66 kg/m^2^, p<0.001), and also reported a higher average number of comorbidities (p<0.001). In all questions related to quality of life, for example general self-perceived health, impairments in daily activities or social contacts, participants with pain rated themselves consistently worser than participants without pain (p<0.001 for all comparisons).

Comparable findings were observed for respondents to the main questionnaire reporting current joint pain of any site. They were significantly older (mean age 62.2±11.8 years versus 57.6±12.1 years) and more often female (p = 0.001). Current joint pain was associated with higher BMI values (mean difference: 1.71, p<0.001), and also with increased comorbidity (p<0.001). Similar to pain of any site, current joint pain was related to lower quality of life ratings (p<0.001 for all comparisons).

**Figure 3:**
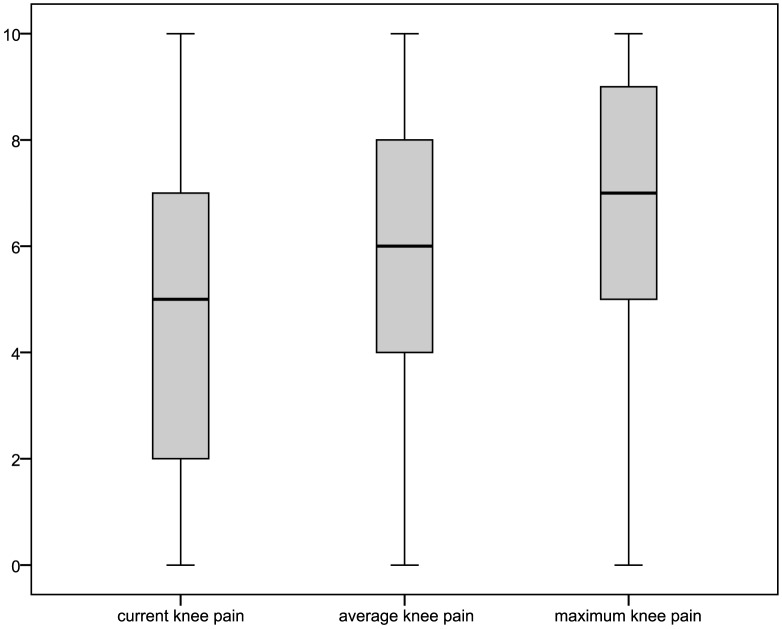
Distribution of pain intensities in respondents to the main questionnaire reporting predominantly knee pain.

**Figure 4:**
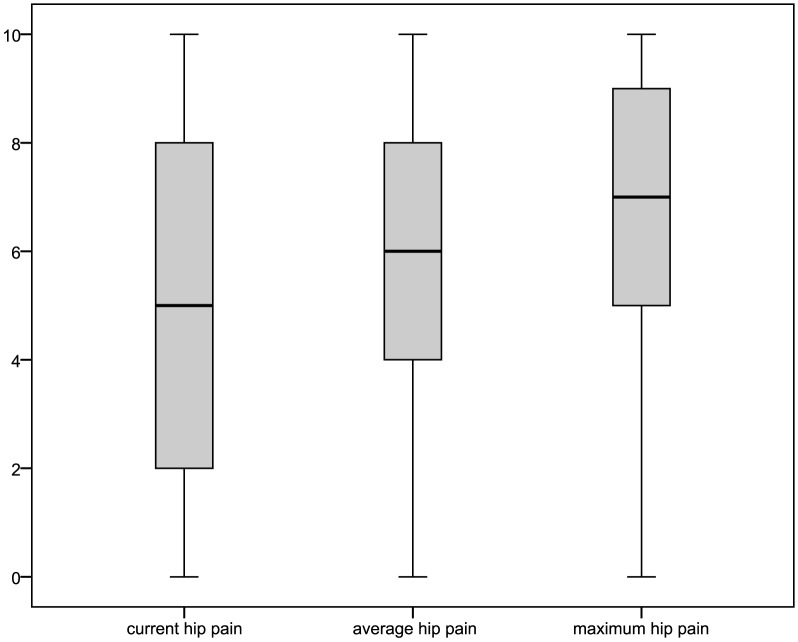
Distribution of pain intensities in respondents to the main questionnaire reporting predominantly hip pain.

**Figure 5:**
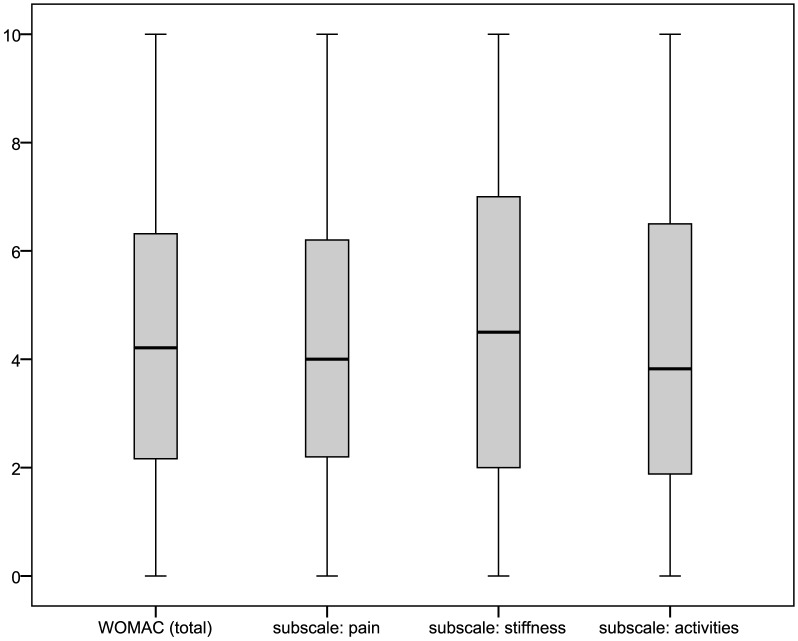
Distribution of the WOMAC total and subscale scores in respondents to the main questionnaire reporting predominantly knee pain.

**Figure 6:**
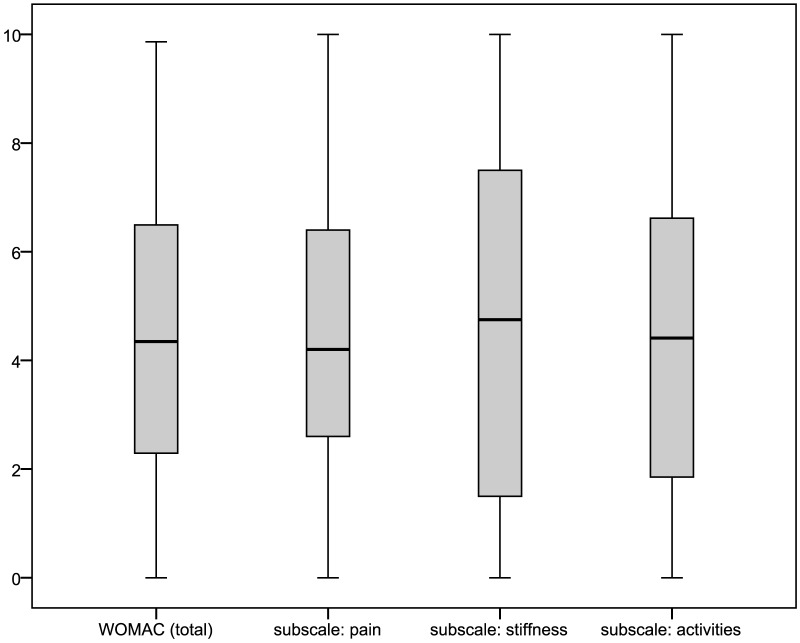
Distribution of the WOMAC summary and subscale scores in respondents to the main questionnaire reporting predominantly hip pain.

For respondents of the main questionnaire reporting joint complaints within a year prior to interview and knee or hip as the joint predominantly affected, pain intensities and the WOMAC could be obtained. Pain intensities for respondents reporting predominantly knee pain, also depicted in figure 3 with box-plots, were: mean 4.86±3.18 (median: 5.0) for current knee pain; 5.62±2.71 (6.0) for average pain; and 6.49±2.79 (7.0) for maximum pain in four weeks prior interview. Values for respondents with predominant hip pain (figure 4) were: mean 5.05±3.07 (median: 5.0) for current hip pain; 5.79±2.54 (6.0) for average pain; and 6.66±2.57 (7.0) for maximum pain in four weeks prior interview. The distribution of WOMAC values (total as well as subscale scores) is shown in figures 5 and 6 for the pain sites knee and hip, respectively. The WOMAC total score, standardized to a 0 – 10 visual-analogue-scale, was mean 4.30±2.58 (median: 4.21) for knee pain and 4.48±2.55 (4.35) for hip pain.

## Discussion

Our study provides current data on pain and musculoskeletal complaints in an urban population of adults aged 40 years and above in Germany. For comparison, current national data are available from the national health surveys performed by the Robert Koch Institute [11], [17], [18], a nationwide survey on pain in the elderly [20], a local telephone survey performed by our study group [21], and further data from some few earlier surveys [22]–[25]. Our estimates are in accordance with these reports. For example, current joint complaints were assessed in 41% to 49% of respondents in the BGS98 [17], and approximately 50% in a survey from Schumacher and Brähler [22]. This fits well with our estimate of roughly 49% for current joint complaints. Hip complaints, prevalent in about 15% of our sample, were reported in about 13% by the BGS98 [17], about 15% by Kohlmann [25], and approximately 16% in our telephone survey [21].

Knee complaints were not estimated by the studies mentioned. Instead, complaints of the leg apart from the hip joint, which was explicitly addressed, were found in 22% and 29%, respectively [17], [25]. This is lower than our estimate of approximately 31% for knee complaints only. If the term “leg complaints” is assumed to summarise all possible joints of a limb including the knee, one would expect a higher prevalence estimate here. It is, however, not likely that participants answer comparably to this rather unspecific site option than to sites explicitly mentioned as in our study. Furthermore, our estimate from the telephone survey of approximately 36% for knee complaints confirms our present estimate [21].

One German survey by Chrubasik and colleagues [23] reported prevalences for pain and joint complaints consistently lower than our estimates and those from other surveys. A six week prevalence for any pain of approximately 47% was assessed, compared to a four week prevalence of 75% in our survey, and 19% and 7% for knee and hip complaints, respectively, in comparison to our estimates of 31% and 15%. The difference is well explained by the fact that Chrubasik et al. asked for prolonged pain [23], thus excluding shorter pain episodes. A mixed urban and rural population and differences in survey methodology may contribute further to the difference.

In comparison to recent European studies, our estimates range within the usual published prevalences. For musculoskeletal complaints and pain, prevalences are reported between roughly 25% and over 75% [1], [3]–[5], [10], [28]. Variability between reports is explained by differences in the definition of musculoskeletal complaints and discrepancies between the populations surveyed. A further source of variability is the wording of survey questions. As has been demonstrated several years ago, even minor differences in wording may lead to different prevalence estimates [29]. In general, expressions from everyday language are broader and lead per tendency to higher prevalence estimates than a more technical wording, especially when demands like a physicians' diagnosis or something likewise are included [5], [11], [13], [28]. Thomas et al., using questions with a wording comparable to that of our study, but applying it to a population aged 50 years and above, assessed a four week prevalence for musculoskeletal pain of 72% [4]. This is higher than our estimate. By contrast, Bergman et al. reported a yearly prevalence as low as 35% [5]. However, they assessed only pain of at least three month duration, which obviously leads to lower pain prevalences.

For site specific complaints, prevalences from European studies range from 15% to 47% for the knee, and 8% to 22% for the hip [4], [5], [9], [10], [12], [13], [28], [30]–[35]. We assessed 31% and 15%, respectively, which is in good accordance with these reports. The predominance of women and the relationship with increasing age has already been reported frequently for both pain and musculoskeletal complaints [3], [12], [28], [31], [36]. The same applies to associations between pain as well as joint complaints and comorbidity, BMI and quality of life [1], [5], [9], [12], [13], [28], [36]. These trends were further confirmed by our data. Exceptions are estimates of the WOMAC, which has rarely been used on the population level. One study [10] using the WOMAC to assess the impact of knee pain in a survey reported lower average values for the total score and all three subscales than we assessed. This, however, is easily explained by the fact that we obtained the WOMAC for a subgroup of participants only, i. e. participants reporting joint complaints.

Some limitations should be considered when interpreting our findings. At first, self–selection of participants, at least to some degree, occurred in our study. We found that survey participants were of higher age and predominantly women, thus possibly reflecting enhanced symptom and disease severity in comparison to the whole population. This effect is only partly diminished by the refusal especially of females of the highest age categories, who likely carry the highest disease burden. Secondly, although the distribution of age and sex among participants is comparable to that of the basic population, differences concerning other characteristics, with socioeconomic status being the most important, can not be ruled out. With the available data, however, we had no opportunity to check or account for this. Despite these aspects, our survey did not show remarkable deviation from characteristics of other national or European surveys, neither qualitatively nor quantitatively [4], [9], [13], [20], [22]–[25]. Differences in survey results are further related to definitions of pain and complaints, the wording of questions and the population surveyed. They do not necessarily threaten the validity of a given survey, yet should be considered when findings of different studies are compared.

## Conclusions

In conclusion, our survey adds important current data to the relatively scarce data base on pain and joint complaints in adults in Germany. Several findings of national as well as European surveys could be confirmed. Given the importance of lower limb function especially for the elderly, the high site specific prevalences of knee and, to a lesser extent, hip complaints underline the necessity to further investigate characteristics and consequences of pain and symptomatic osteoarthritis of the major lower limb joints in adults in Germany.
